# TRIM study protocol - a prospective randomized multicenter Trial to assess the Role of Imaging during follow-up after radical surgery of stage IIB-C and III cutaneous malignant Melanoma

**DOI:** 10.1186/s12885-020-07632-4

**Published:** 2020-12-07

**Authors:** Ylva Naeser, Hildur Helgadottir, Yvonne Brandberg, Johan Hansson, Roger Olofsson Bagge, Nils O. Elander, Christian Ingvar, Karolin Isaksson, Petra Flygare, Cecilia Nilsson, Frida Jakobsson, Olga del Val Munoz, Antonis Valachis, Malin Jansson, Charlotte Sparring, Lars Ohlsson, Ulf Dyrke, Dimitrios Papantoniou, Anders Sundin, Gustav J. Ullenhag

**Affiliations:** 1 Department of Immunology, Genetics and Pathology, Uppsala University, Rudbeck laboratory, 75185 Uppsala, Sweden; 2 Department of Oncology, Uppsala University Hospital, entrance 101, 1tr, 75185 Uppsala, Sweden; 3 Department of Oncology, Karolinska University Hospital Solna, 17164 Solna, Sweden; 4 Department of Oncology-Pathology, Karolinska Institutet, 17177 Stockholm, Sweden; 5 Sahlgrenska Cancer Center, Department of Surgery, Institute of Clinical Sciences, Sahlgrenska Academy, University of Gothenburg, Gothenburg, Sweden; 6 Department of Surgery, Sahlgrenska University Hospital, 41345 Gothenburg, Sweden; 7 Wallenberg Centre for Molecular and Translational Medicine, University of Gothenburg, Gothenburg, Sweden; 8 Department of Oncology and Department of Biomedical and Clinical Sciences, Linköping University, 58185 Linköping, Sweden; 9 Department of Surgery, Clinical Sciences, Lund University, BMC F12, 22184 Lund, Sweden; 10 Department of Surgery, Central Hospital Kristianstad, 29133 Kristianstad, Sweden; 11 Department of Clinical Sciences, Surgery, Lund University, BMC F12, 22184 Lund, Sweden; 12 Department of Oncology, Sundsvall County Hospital, Lasarettsgatan 21, 856 43 Sundsvall, Sweden; 13 Department of Oncology, Hospital of Västmanland Västerås, 72189 Västerås, Sweden; 14 Department of Oncology, Örebro University Hospital, 70185 Örebro, Sweden; 15 Department of Oncology, Gävle County Hospital, 80187 Gävle, Sweden; 16 Department of Oncology, Faculty of Medicine and Health, Örebro University, 70182 Örebro, Sweden; 17 Department of Surgical and perioperative sciences, Umeå University and Umeå University Hospital, 90185 Umeå, Sweden; 18 Department of Dermatology, Skaraborg County Hospital, 54185 Skövde, Sweden; 19 Department of Surgery, Karlstad County Hospital, Rosenborgsgatan 9, 65230 Karlstad, Sweden; 20 Department of Surgery, Falun County Hospital, 79182 Falun, Sweden; 21 Department of Oncology, Ryhov County Hospital, 55185 Jönköping, Sweden; 22 Department of Surgical Sciences Radiology & Molecular Imaging, Uppsala University, 75185 Uppsala, Sweden

**Keywords:** Cutaneous malignant melanoma, Follow-up, FDG-PET/CT, CT

## Abstract

**Background:**

The incidence of cutaneous malignant melanoma (CMM) is increasing worldwide. In Sweden, over 4600 cases were diagnosed in 2018. The prognosis after radical surgery varies considerably with tumor stage. In recent years, new treatment options have become available for metastatic CMM. Early onset of treatment seems to improve outcome, which suggests that early detection of recurrent disease should be beneficial. Consequently, in several countries imaging is a part of the routine follow-up program after surgery of high risk CMM. However, imaging has drawbacks, including resources required (costs, personnel, equipment) and the radiation exposure. Furthermore, many patients experience anxiety in waiting for the imaging results and investigations of irrelevant findings is another factor that also could cause worry and lead to decreased quality of life. Hence, the impact of imaging in this setting is important to address and no randomized study has previously been conducted. The Swedish national guidelines stipulate follow-up for 3 years by clinical examinations only.

**Methods:**

The TRIM study is a prospective randomized multicenter trial evaluating the potential benefit of imaging and blood tests during follow-up after radical surgery for high-risk CMM, compared to clinical examinations only. Primary endpoint is overall survival (OS) at 5 years. Secondary endpoints are survival from diagnosis of relapse and health-related quality of life (HRQoL). Eligible for inclusion are patients radically operated for CMM stage IIB-C or III with sufficient renal function for iv contrast-enhanced CT and who are expected to be fit for treatment in case of recurrence. The planned number of patients is > 1300. Patients are randomized to clinical examinations for 3 years +/− whole-body imaging with CT or FDG-PET/CT and laboratory tests including S100B protein and LDH. This academic study is supported by the Swedish Melanoma Study Group.

**Discussion:**

This is the first randomized prospective trial on the potential benefit of imaging as a part of the follow-up scheme after radical surgery for high-risk CMM.

**Results:**

The first patient was recruited in June 2017 and as of April 2020, almost 500 patients had been included at 19 centers in Sweden.

**Trial registration:**

ClinicalTrials.gov, NCT 03116412. Registered 17 April 2017, https://clinicaltrials.gov/ct2/show/study/NCT03116412

## Background

The incidence of cutaneous malignant melanoma (CMM) increases rapidly in many countries. In Sweden there was a 35% increase in incidence between 2013 and 2018, from 3400 to 4600 cases [1]. According to the 2016 annual report from the Swedish Cancer Society, the number of cases are expected to multiply during the next decades [2]. Hence, the number of patients requiring follow-up after surgery for CMM will steadily grow in the future.

The reasons for follow-up include:

- To early detect and treat locoregional and distant relapses in order to improve survival.
- To early detect a new primary CMM.
- To inform, educate and support the patient.

The potential value of follow-up programs after radical surgery for CMM has been a matter of debate for many years, and with little scientific evidence in support. A Dutch trial, randomizing stage IB-IIC patients between conventional and stage-adjusted follow-up by physical examinations in the standard arm according to Dutch guidelines and less frequent physical examinations in the experimental arm. Patient-reported outcome measures (PROMs) were completed at 0, 1 and 3 years after diagnosis. Primary outcome was patients´ well-being and secondary outcomes were recurrences and melanoma-related deaths. One hundred and eighty patients were included and the results were published this year. Analysis three years after diagnosis showed no difference in RFS (recurrence-free survival) or DFS (disease-free survival) [3]. Other studies, published a decade ago, have reported relatively low predictive value of the elevated tumor marker, S100B protein in plasma to detect relapse [4, 5].

An important question is whether imaging should be a part of the follow-up program for patients who have undergone surgery for high-risk CMM. Some studies have investigated the value of follow-up including imaging in this patient group. The MSLT-2 study, in a large number of patients (*n* = 1934), showed in 2017 that repeated controls by ultrasound could replace lymph node dissection in case of a positive sentinel node biopsy (SNB) [6]. However, until now, no randomized study investigating the benefit of whole-body imaging has been conducted. In 2017 the results of an American retrospective study were presented and included 247 CMM patients, 27% followed with clinical examinations only and the remaining 73% underwent a combination of clinical examinations and imaging. The mode of imaging was heterogenous and was limited to chest X-ray for stage IIA/IIB patients. Eighty-seven percent of stage IIC/III patients underwent at least two series of whole body PET/CT or whole body CT plus MRI of the brain. The recurrence rate for stage IIC/III was 23% at a median follow-up time of 31 months. Fifty percent of recurrences in this group were detected by imaging in asymptomatic patients [7].

In a retrospective study of 173 CMM patients from three centers in the UK, CT or FDG-PET/CT plus MRI of the brain were performed according to a UK consensus stipulating repeated imaging up to five years for high-risk patients. Forty-seven per cent had relapsed at a median follow-up time of 23 months, the majority of which (66%) was detected by imaging. Median time to recurrence was ten months. Forty-five per cent of relapsing patients underwent surgery and median OS for this subgroup has not been reached. Median OS for patients who received systemic therapy was 13 months [8]. An Australian study, published in the same year (2018), had a similar approach which but resulted in fewer detected relapses than in the previous study. In a PET database, 170 stage III melanoma patients were identified who regularly had undergone routine FDG-PET/CT +/−MRI of the brain. Melanoma relapse was detected in 38% and mainly (69%) in asymptomatic patients. No relapses were identified by clinical examination without first being reported by patients. Fifty-two percent of relapsing patients underwent surgery. Median follow-up time was 47 months [9].

In addition, a few prospective non-randomized studies have also been conducted. In a Spanish study, published in 2016, almost half of 115 recorded relapses in 290 patients were detected by imaging [10]. In a large German study on patients with all stages of CMM (*n* = 2008), a follow-up program including frequent imaging examinations was prospectively applied. The authors presented the results in 2008 and concluded that the relapses mainly occurred during the first years after radical surgery and earlier in stage III disease compared to stages I-II [11].

The findings in these studies suggest that if a follow-up program is introduced, it should be more intense during or limited to the first years after diagnosis. The observation that more than half of new primary CMMs are detected during the first two years following surgery further indicates that follow-up, if applied, should focus on this time period [11–13]. In a recent Australian cohort study, also with prospective approach, stage III CMM patients (*n* = 154) underwent either CT or FDG-PET/CT at baseline and at least twice annually thereafter. Median follow-up time was 85 months and distant metastasis was identified in 13%. However, 124 out of 1022 examinations were false-positive or showed incidental findings, resulting in unnecessary invasive procedures in 15 patients. The authors concluded in their publication last year that false-positive results and incidental findings occurred in at least half of all patients with substantial additional demands on healthcare resources [14]. This study underlines the costs as a major drawback of intense follow-up programs with imaging. In the choice of imaging, FDG-PET/CT is more expensive than CT, and not as widely available, but covers the whole body and offers slightly better sensitivity than CT. Another difference between the two methods is that a CT scan has a higher predictive value than a PET/CT scan in identifying metastases to the liver and lung. For detection of brain metastases, MRI is better than CT, even though the difference is small when contrast-enhanced high resolution CT protocol is applied as in our study. MRI is associated with long scan times, contraindications (the presence of metal implants) and is more costly and less available than CT [15]. Another negative effect of routine imaging (except for MRI) is the irradiation exposure to the patient, which should not be neglected even though the doses are clearly lower with modern scanners [16].

In recent years, new treatment options have become available for metastatic CMM; immunotherapy and targeted therapy. The introduction of novel drugs emphasizes the need to develop optimal follow-up programs for early detection of recurrent disease, since early onset of treatment seems to improve outcome [17, 18].

According to the Swedish national guidelines [19], blood tests or imaging are not parts of the follow-up schedule for CMM. The current recommendation is clinical examinations only for three years for stage IB-III, except in patients with a positive SNB when lymph node dissection is not performed. These patients for three years regularly undergo ultrasound of the positive regional lymph node region [6]. Despite lack of evidence, there is an international tendency for more frequent imaging, such as in the Nordic countries, except Sweden, where FDG-PET/CT is performed regularly up to five years [20, 21].

In summary, there is lack of evidence regarding the potential benefits of follow-up schedules, and even less so concerning the choice of methods, or schedule intensity to apply. The aim of this paper is to present the study design of the randomized TRIM study, evaluating the effects of adding whole-body imaging and laboratory tests to clinical examinations according to Swedish national guidelines.

## Methods

### Study design

The TRIM study is a prospective randomized multicenter trial in which patients with stage IIB-C or stage III CMM are randomized to follow-up for three years by clinical examination alone according to current Swedish national guidelines +/− whole-body imaging with CT or FDG-PET/CT and laboratory tests including S100B and LDH. All patients are followed with yearly updates of survival for 5 years.

### Endpoints

Primary endpoint is OS at five years.

### Secondary endpoints

The secondary endpoints are survival from diagnosis of relapse and health related quality of life (HRQoL).

### Additional evaluations

- Disease-free survival (DFS).
- The number of imaging examinations in addition to the scheduled.
- Health economics.
- The value of blood tests to detect recurrence in comparison to imaging examinations.

### Study population

The study population includes both patients treated for primary melanoma and patients with a recurrent resectable stage III disease. The patients are allowed to receive post-operative adjuvant systemic treatment and / or local radiotherapy according to Swedish national guidelines. In these guidelines it’s recommended that eligible patients are given the opportunity to participate in the study. This helps to achieve adequate participant enrollment. Patients are recruited from all over Sweden at approximately 20 centers, including all university hospitals.

### Key inclusion criteria

- Age above 18 years.
- Signed and dated written informed consent.
- Radical surgery for stage IIB-C or III CMM.
- Sufficient renal function for iv contrast-enhanced CT.

### Key exclusion criteria

- The patient is assessed as unfit to receive treatment in case of recurrence.
- Life-expectancy less than 2 years due to concurrent disease.
- Inability to comply with the follow-up programs.
- Participation in other clinical trials interfering with the control-program.

### Study procedure and randomization

**Fig. 1:**
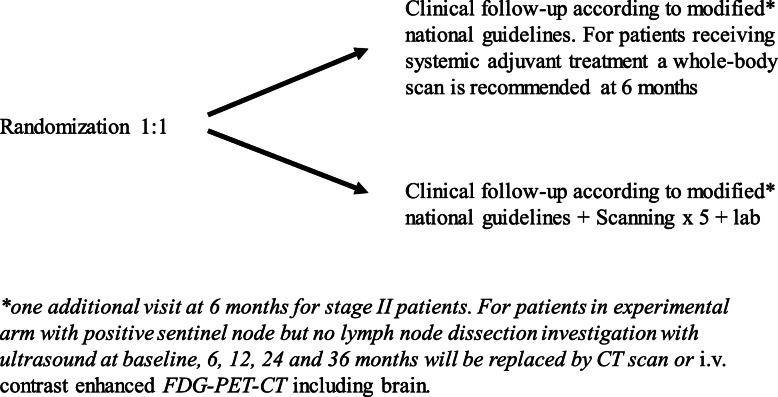
Overall trial design

Written informed consent must be obtained within 8 weeks before randomization. The overall trial design is shown in Fig. 1. The patient must be randomized within 8 weeks after final surgery, i.e. wide excision + sentinel node biopsy or lymph-node dissection.

A baseline visit 1–8 weeks after the final surgery is scheduled for study information, inclusion and randomization. The national schedule for follow up visits is applied for all patients in the study, with exception for an additional visit at month 6 for stage IIB-C patients.

Randomization 1:1 is performed in the Electronic Data Capture (EDC) system (Viedoc) at each site and stratified according to tumor stage and radiological assessment method. The patients are randomized between:

- *1: Low frequency (standard arm) follow-up regimen*

Follow-up for 3 years according to Swedish national guidelines with clinical examinations every 12 months for stage II and every 6 months for stage III patients. Ultrasound of regional lymph nodes is recommended every 6 months for patients with a positive SNB who have not undergone lymph node dissection. For patients receiving systemic adjuvant treatment, a whole-body CT or FDG-PET/CT is recommended at 6 months.

- *2: High frequency (experimental arm) follow-up regimen*

The follow-up is similar to the standard arm, except for regional ultrasound which will only be performed at month 18 and 30 for stage III patients with a positive SNB but without subsequent lymph node dissection. A detailed flow chart for the experimental arm is shown in Table 1.

- Laboratory tests (creatinine, S100B protein, ALP, LDH, AST and ALT) at baseline, 6, 12, 24 and 36 months.
- Whole-body imaging at baseline, 6, 12, 24 and 36 months. The modality of imaging (CT or FDG-PET/CT) is chosen at baseline by the investigator and is kept the same for all examinations.
- CT of the thorax-abdomen and brain must be performed with iv contrast enhancement. In case of a positive SNB but without subsequent lymph node dissection, CT must include locoregional lymph nodes.
- Whole-body FDG-PET/CT includes the brain to the proximal thighs and the concomitant CT is performed according to a diagnostic examination protocol with iv contrast-enhancement. If the melanoma was situated on a leg, the FDG-PET/CT field-of-view must also include the primary site.

**Table 1:** Flow chart for the experimental study arm

Assessments:	Baseline	6 months +/− 28 days	12 months +/− 28 days	18 months +/− 28 days	21 months +/− 28 days	24 months +/− 28 days	30 months +/− 28 days	36 months +/− 28 days	Year 4 (OS only)	Year 5 (OS only)
	1–8 weeks after final surgery(a)			Visit applicable for stage III patients only	Letter contact		Visit applicable for stage III patients only			
Informed consent (within 8 weeks prior to randomization) (b)	X									
Inclusion/exclusion criteria	X									
Demographics	X									
Cancer/treatment history (c)	X									
Physical examination (d)	X	X	X	X (d)		X	X (d)	X		
Biochemistry (e)	X	X	X			X		X		
CT scan thorax/abdomen/brain or iv contrast enhanced whole-body FDG-PET/CT including brain (f)	X (i)	X	X			X		X		
Ultrasound of affected lymph node location (g)				X (g)			X (g)			
HRQoL (h)	X		X		X	X		X		

^a^An imaging examination carried out before but within two months of randomization is adequate as baseline assessment for both groups under the prerequisite that the same method is used for future examinations. Final surgery is defined as wide excision and/or sentinel node biopsy or lymph-node dissection.

^b^Written informed consent must be obtained before any study-specific screening procedures are performed.

^c^Includes thickness of primary tumour, number of examined nodes/nodes with metastases, TNM staging, postoperative treatment (i.e. adjuvant radiotherapy, adjuvant systemic treatment).

^d^To be performed according to clinical routines. Months 18 and 30: Visit applicable for stage III patients only. FU year 4 and 5 is for overall survival only. If visit at the clinic is not planned, a review of medical records is sufficient.

^e^Biochemistry: s-creatinine, S-S100B, ALP, LDH, AST and/or ALT.

^f^The mode of imaging (CT scan of the lungs, abdomen and brain or i.v. contrast enhanced whole body FDG-PET-CT including brain) is chosen by the investigator at baseline and the same mode is used for the subsequent examinations.

^g^To be done shortly before the scheduled visits, that are not preceded with whole body imaging examination, for stage III patients who have not had lymph node dissection.

^h^HRQoL (EORTC QLQ-C30 and HAD) will be carried out at sites with an oncologist as PI. The assessments will be performed at the study center, just before the study visits, preferably on a web-based device (if not available, paper formula is accepted). At 21 months the HRQoL will be completed remotely between on-site visits.

^i^If not performed within 8 weeks before randomization, it should be done within 28 days post-randomization at the latest.

### Extra visits and investigations

Extra diagnostic evaluation such as imaging and laboratory tests are allowed for all subjects presenting signs and/or symptoms. Patients in both groups are instructed to contact their study center if they experience any symptoms suspicious of recurrence. Additional controls are registered in the eCRF under “Extra visits”.

### Health related quality of life (HRQoL)

For logistical reasons, HRQoL assessments are only carried out at sites with an oncologist as principal investigator (PI). The assessments are scheduled at baseline and at visits at 12, 24 and 36 months. The baseline assessment is performed after the informed consent procedure but before randomization. Thus, the patient should be unaware of the result of randomization when completing the first HRQoL-questionnaire. The assessments are made at the study center, just before the study visits, preferably on a web-based device administered by a research nurse. Validated HRQoL-instruments are used (EORTC QLQ-30 and HAD-scale). Additionally, at month 21, a letter is sent to the patients asking them to complete an extra HRQoL questionnaire at home, including a prepaid return envelope.

### Questionnaires

The EORTC Quality of Life Core Questionnaire (EORTC QLQ-C30), Version 3 is a cancer-specific questionnaire consisting of 30 items including five functional scales (physical-, role-, emotional-, cognitive-, and social functioning), three multi-item symptom scales (fatigue, pain, nausea and vomiting), five single items (dyspnea, loss of appetite, constipation, insomnia, diarrhea, financial difficulties related to the disease) and a global quality of life scale [22]. The responses are scored with 4-point scales ranging from 1 (“Not at all) to 4 (“Very much”), with the exception of the two items assessing global quality of life which are scored on a 7-point scale ranging from 1 (“Very poor”) to 7 (“Excellent”). The time frame is “during the previous week”. Reference values from the Swedish population are available [23].

The Hospital Anxiety and Depression Scale (HAD-scale) is developed to assess anxiety and depressive symptoms in somatically ill patients [24]. It consists of 14 items, 7 assessing anxiety (HAD- A) and 7 assessing depression (HAD-D). The presence of problems during the preceding week is rated on a four- graded scale from 0 (“No problem”) to 3. The scores for each subscale are summed, giving a maximum of 21. Two cut-off points have been suggested: 0–7 no problems of clinical relevance (non-cases), 8–10 cases that warrant further psychiatric investigation (possible cases), and ≥ 11 clinical levels of anxiety/depression (probable cases). The HAD-scale has been widely used and the Swedish version has been validated in patients with CMM [25] and in breast cancer patients [26].

### Duration

Follow-up according to protocol will continue for three years if no signs of recurrent disease. In case of loco-regional recurrent disease treated with radical surgery, the patient restarts the follow-up program in the same study arm if all inclusion criteria for the study still are met. All patients will be followed for survival for 5 years with survival updates at 4 and 5 years.

Discontinuation criteria

1. Withdrawal of consent.
2. Inappropriate enrollment.
3. Recurrent stage IV or unresectable stage III CMM.
4. Subject unable or unwilling to continue follow-up program according to protocol.
5. Conditions leading to frequent radiological assessments other than stipulated by the TRIM study protocol, for example enrollment in other clinical studies or other malignancies.

### Statistical considerations

The sample size is based on the primary endpoint OS at 5 years.

Based on data from the Swedish Melanoma Registry, the following numbers of patients diagnosed and classified according to AJCC7 (version 7) are expected:

- Stage IIB-C: 325 patients/year with a risk of recurrence during the first 3 years of 20–30%.
- Stage III: 350 patients/year with a risk of recurrence during the first 3 years of 50–60%.

The 5-year mortality for the entire group is estimated at 40% and we aim to detect a difference in hazard ratio for death of at least 20%. With a risk of type 1 error of 5% and type 2 error of 15%, 573 patients should be randomized to each group. With an expected dropout rate of about 10%, the planned number of randomized patients should be 1300.

However, with the introduction of systemic adjuvant treatment for stage IIIB-D patients in early 2019, less relapses are expected to occur in this subgroup. Impressive long time results for systemic treatments in advanced disease have also been presented [27, 28]. Hence, more patients than originally planned need to be included to meet the statistical requirements. Therefore, updated power calculations taking these factors into consideration are planned when final OS results for the pivotal adjuvant studies (COMBI-AD, CheckMate 238 and KEYNOTE-054) have been compiled (www.clinicaltrials.gov; NCT01682083, NCT02388906 and NCT02362594). Since the final OS analysis for the COMBI-AD study is planned in the end of 2021, the final OS results of CheckMate 238 will most likely also be presented next year and the final OS analysis for the Keynote-54 study is expected in less than three years, we plan to conduct new power calculations in autumn 2023. We expect that patient recruitment has not ended by then.

Results will be evaluated both on an intention-to-treat basis and per protocol. Patients who withdraw their informed consent remain in their allocation group when evaluated on the intention-to-treat basis, and are excluded when evaluated on the fulfilled protocol basis.

Survival data will be analyzed according to the Kaplan-Meier method and comparison between groups will be performed with the log-rank method. Binominal data will be analyzed with Chi-square statistics and continuous data with Mann-Whitney U test. Level of significance two-sided *p* < 0.05.

#### Health related quality of life (HRQoL)

The values on the EORTC QLQ-C30 subscales will be calculated and transformed to 100-points scales according to guidelines. The two subscales of the HAD-scale will be calculated as in the original publication, and suggested cut-offs will be implemented, making it possible to compare proportions of patients using the Chi2-test. HRQoL will be analyzed by linear regression models, including both randomization arms and adjusted for sex, age and baseline values for the studied scales.

Only persons at the main study center with defined roles will have access to the final trial data set.

### Ethical considerations

Patients in both groups will receive follow-up according to standard of care in Sweden today. Thus, we consider there is no risk for inadequate care by inclusion in the TRIM study. All participants must give written informed consent for participation. This can be withdrawn at any time. Patients declining to participate or who withdraw from the study will be followed according to the national guidelines i.e. the same follow-up as the study control group. The principles in the Declaration of Helsinki will be met.

It is uncertain whether imaging during follow-up is of benefit for the patients. The imaging examination schedule in this study is unlikely to harm the individual patient, although the radiation dose should not be neglected.

A negative impact on the patient’s well-being in the waiting period after the imaging examination, before he/she is informed of the results, cannot be ruled out. Furthermore, inconclusive imaging results, sometimes leading to further investigations, i.e. biopsies and/or a repeated imaging examination are likely to cause distress and reduced HRQoL. Therefore, we consider it important to assess the impact of the follow-up procedures in the TRIM-study.

## Discussion

The incidence of CMM is rapidly increasing in many countries and hence the number of patients who will be subject to follow-up is growing. Follow-up programs usually aim to support and educate the patients and to improve outcome by earlier detection of recurrent disease, both locoregional and distant. Early detection of recurrence is proposed to be beneficial by increasing the chance of radical resection or enabling an early onset of systemic treatment. Due to lack of reliable data it is, however, unclear if scheduled follow-up visits lead to earlier detection of localized disease. One exception is when regional ultrasound is performed in patients with a positive SNB, but no benefit in survival was demonstrated [6]. In fact, the patients usually detect their relapses themselves despite regular controls [29].

In earlier studies the majority of relapses occurred during the first years after primary surgery, indicating that follow-up is most appropriate in the first few years after diagnosis. However, little is known about the potential OS benefit in finding recurrent metastatic disease early, not the least in the modern era with potent systemic melanoma treatments available. As a consequence, there are great variations between national guidelines regarding the recommendations for follow-up after surgery for high-risk CMM. An increasing number of countries have implemented whole-body FDG/PET-CT or CT at various frequencies and duration, as part of their routine follow-up programs in spite of lack of evidence from randomized studies. Intense follow-up programs require resources which are expensive and might negatively affect the patients´ HRQoL.

The importance of not introducing a more intense follow-up program based on mere assumptions of benefit is underlined by the results of the COLOFOL study. In this study, additional CT examinations after curative surgery for colorectal cancer did not result in a significant reduction in 5-year overall mortality or colorectal cancer–specific mortality [30].

In conclusion, the impacts of high-volume follow-up programs including imaging after radical surgery for CMM on OS are not sufficiently studied to create evidence-based guidelines. Despite this, the Current Oncological Practice (COP) is moving towards high intensity follow-up programs while a Minimal Acceptable Practice (MAP) might be just as good. It is therefore of uttermost importance to perform a large controlled trial, randomizing patients to control programs with or without imaging. The TRIM study aims to include at least 1300 patients and is the first randomized trial to investigate the potential benefit of imaging assessments as part of the follow-up program after radical surgery of CMM and to explore their impact on the patients´ HRQoL. A first HRQoL data analysis is planned when 100 patients in each study arm have completed baseline and first year HRQoL assessments which is expected in early spring 2021. The results of the TRIM study will most likely impact future guidelines for follow-up of high-risk CMM patients.

### Trial status

**Fig. 2:**
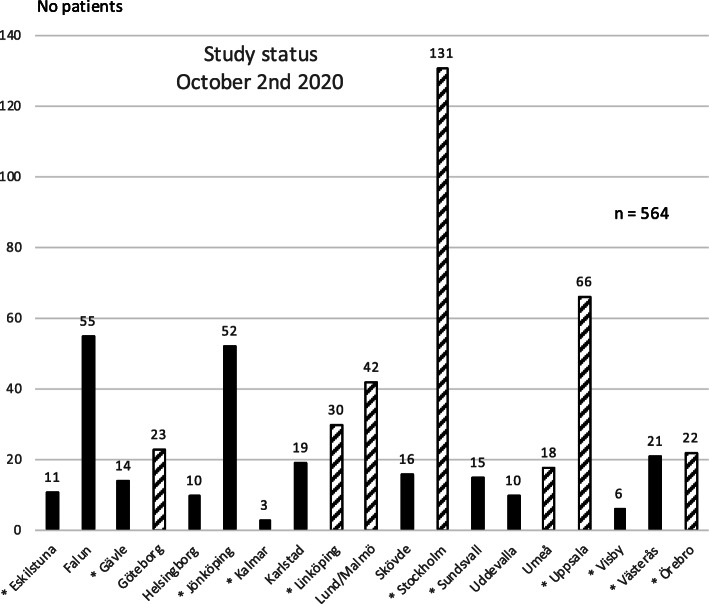
Recruitment status for individual centers. Striped bars represent university hospitals. Oncology sites are marked with a star (*)

The first patient was recruited in June 2017. On the 2nd of October 2020, 564 patients had been recruited at 19 centers in Sweden as shown in Fig. 2. Three hundred and sixty-eight patients out of 564 were included at oncology sites. Recruitment is ongoing. Latest protocol version and date: 1.4, 2018-12-11.

The inclusion rate differs substantially between centers. One major reason is the large differences in target populations between centers. However, some centers have a large potential to recruit more patients and the various reasons for suboptimal inclusion rates are important to address for the study team. Obstacles so far have consisted of:

- Long waiting times for pathology reports after wide excision +/− SNB results at some sites resulting in missing deadline for study inclusion. These waiting times have improved over time and to further diminish this problem, time from final surgery to possible study inclusion was extended from six to eight weeks.
- In some regions, stage II patients have not been discussed at a multidisciplinary conference and therefore, all surgeons have not referred these patients to the regional study center for study participation. This has improved and it is also possible to start up satellite centers.

Very few patients have declined participation in the TRIM study when offered to participate.

Strategies to improve adherence to the protocol, besides monitoring, include regular newsletters to the study centers and continuous support mainly by email.

## Data Availability

Not applicable since the trial is ongoing and no results are reported. The results of the study will be presented in future publications.

## Abbreviations

CMM: Cutaneous malignant melanoma
OS: Overall survival
SNB: Sentinel node biopsy
HRQoL: Health related quality of life
DFS: Disease free survival
